# The role of anakinra in the modulation of intestinal cell apoptosis and inflammatory response during ischemia/reperfusion

**DOI:** 10.3906/sag-2008-258

**Published:** 2021-08-30

**Authors:** Muhammed KANDEMİR, Necdet Fatih YAŞAR, Mete ÖZKURT, Rumeysa ÖZYURT, Nuriye Ezgi BEKTUR AYKANAT, Nilüfer ERKASAP

**Affiliations:** 1 Department of General Surgery, Faculty of Medicine, Osmangazi University, Eskişehir Turkey; 2 Department of Physiology, Faculty of Medicine, Eskisehir Osmangazi University, Eskisehir Turkey; 3 Department of Physiology, Faculty of Medicine, Kütahya University, Kütahya Turkey; 4 Department of Histology and Embryology, Faculty of Medicine, Atılım University, Ankara Turkey

**Keywords:** Ischemia reperfusion injury, interleukin-1 receptor antagonist, anakinra

## Abstract

**Background/aim:**

Even though interleukin-1 receptor antagonist, IL-1Ra, is used in certain inflammatory diseases, its effect on ischemia-reperfusion injury is a current research topic. We aimed to investigate the protective effects of anakinra, an IL-1Ra, on the I/R induced intestinal injury.

**Materials and methods:**

The rat model of intestinal ischemia-reperfusion was induced. Rats were randomized into 4 groups: (group 1) control group, (group 2) I/R group, (group 3 and 4) treatment groups (50 mg/kg and 100 mg/kg, respectively). Gene expressions of caspase-3, TNF-α, IL-1α, IL-6, and apoptotic cells in tissue samples were evaluated by PCR and TUNEL methods, respectively. Plasma levels of superoxide dismutase (SOD), catalase (CAT), and malondialdehyde (MDA) were studied by the ELISA method and tissue samples were examined histopathologically as well.

**Results:**

Anakinra inhibited the expression of IL-1α, IL-6, and TNF-α and decreased the SOD, CAT, and MDA caused by ischemia-reperfusion injury in both treatment groups. Caspase-3 expression and TUNEL-positive cell number in treatment groups were also less. Histopathologically, anakinra better preserved the villous structure of the small intestine at a dose of 100 mg/kg than 50 mg/kg.

**Conclusion:**

Anakinra decreased the intestinal damage caused by ischemia-reperfusion and a dose of 100 mg/kg was found to be histopathologically more effective.

## 1. Introduction

Intestinal ischemia occurs when intestinal blood flow rapidly decreases following certain clinical situations, such as mesenteric vascular occlusion, low cardiac output, and sepsis, and rapidly may develop into a life-threatening abdominal emergency [1]. Reperfusion is required to salvage the ischemic tissue and can paradoxically exacerbate tissue injury by inducing the production of reactive oxygen species, proinflammatory cytokines, and activation of the complement system [2]. 

Activated macrophages during reperfusion secrete various proinflammatory cytokines such as TNFα, IL-1, and IL-6 which promote proliferation, production of oxygen-derived free radicals, and chemotaxis [3]. Interleukin-1 (IL-1), one of these proinflammatory cytokines, plays a major role in the orchestration of mediator responses to ischemia-reperfusion (I/R) injury by increasing the expression of additional cytokines and chemokines and can provoke further inflammation [4]. 

Although blockage of IL-1 is used in certain inflammatory diseases such as rheumatoid arthritis, it is also a current research area for I/R treatment [5]. Anakinra is a recombinant, nonglycosylated synthetic form of the human interleukin-1 receptor antagonist (IL-1Ra) which antagonizes competitively the biological effects of IL-1 and decreases the levels of reactive oxygen species by inhibiting the effects of other inflammatory factors [6]. By these effects, it may protect against I/R injury. Previous studies demonstrated that IL-1Ra possesses certain therapeutic effects on the I/R injury in the brain, ovary, and other organs [7,8]. Even though there have been many reports indicating that various types of anti-inflammatory agents can attenuate intestinal I/R injury, it cannot be fully controlled and its prognosis has not dramatically changed. Based on this evidence, in the present study, we investigated the possible protective effects of anakinra, an IL-1Ra, on the I/R induced intestinal injury.

## 2. Materials and methods

### 2.1. In vivo experimental model and treatments

This study was approved by the Local Ethical Committee of Animal Experiments of Eskisehir Osmangazi University (protocol no. 2018-2438). In this study, 32 adult male Sprague-Dawley rats weighing 200–250 g were used. All animals were kept in a temperature-controlled room (22 °C ± 2 °C) with a 12-h light-dark cycle and had free access to water and standard laboratory chow.

Rats were randomized into four groups: (Group 1) control group, the superior mesenteric artery was exposed and intestinal were collected with no manipulation; while in the I/R group (Group 2), the superior mesenteric artery (SMA) was nipped at its root to interrupt blood supply for 1 h and then the arterial nipper was taken off to permit reperfusion of the bloodstream for 2 h. The procedures for groups 3 and 4 were the same as that in the I/R group and 50 mg/kg and 100 mg/kg of anakinra (Kineret, Swedish Orphan Biovitrum, Stockholm, Sweden) was injected intraperitoneally 1 h before the operation, respectively. The intestinal samples were taken off at the 5 cm site distant from the ileocecal junction after the completion of the experiment, along with blood samples.

### 2.2. RNA extraction and quantitative reverse transcription PCR 

Total RNA was isolated from terminal ileum tissues using the GeneJet RNA Purification Kit (Thermo Scientific, USA). The concentration and purity of the RNA were measured using a NanoDrop 1000 (Thermo Scientific, USA). Isolated RNA samples were converted to complementary DNA (cDNA) using the RevertAid First Strand cDNA Synthesis Kit (Thermo Scientific, USA) at 42 °C for 60 min and 70 °C for 5 min. cDNA samples were stored at –80 °C until analysis. Caspase-3, TNF-α, IL-1α, IL-6 gene expressions were measured with SYBR Green qPCR Kit (Thermo Scientific, USA). cDNA synthesis was verified by detection of the β-actin transcript, which was used as an internal control. Relative differences in expression were determined using the comparative threshold cycle (2^-ΔΔCt^ ) method.

### 2.3. Measurement of SOD, CAT, and MDA from blood plasma by ELISA method

After the surgical procedures, intracardiac blood samples were taken from all rats. The blood samples were centrifuged at 4100 g for 15 min. Plasmas remaining in the upper phase were taken into sterile tubes through a pipette. In plasma samples, superoxide dismutase, catalase, and malondialdehyde levels were measured.

### 2.4. Malondialdehyde (MDA) measurement 

The protocol proposed by Ohkawa et al. in 1979 was used for MDA measurement in plasma [9]. Tetrametoxypropane was used as standard. In the spectrophotometer, it was calculated by measuring the colored compounds at 532 nm wavelength by reacting MDA with thiobarbutyric acid (TBA). MDA concentrations in plasma were determined using commercially available TBARS kits as recommended by the manufacturers (Awareness Technology, Inc. Martin Hwy. Palm City, USA). Results are given in µmol/L.

### 2.5. Catalase (CAT) activity measurement 

The protocol proposed by Aebi in 1984 was used for CAT measurement in plasma [10]. The decomposition ratio of H_2_O_2_ (hydrogen peroxide) in erythrocyte hemolysate at 25 ºC was measured spectrophotometrically at 240 nm for 30 s. Catalase activity in plasma was determined using a commercially available kit as recommended by the manufacturers (Cayman Chemical, Ann Arbor, Michigan, USA). Absorbance was measure by an ELISA reader (Awareness Technology, Inc. Martin Hwy. Palm City, USA). Results are given in nmol/min/mL.

### 2.6. Superoxide dismutase (SOD) activity measurement 

SOD activity measurement in plasma was made by measuring at a wavelength of 505 nm the intensity of the violet-colored formazan dye created by reacting with iodonitrotetrazolium. SOD activity in plasma was determined using a commercially available kit as recommended by the manufacturers (Cayman Chemical, Ann Arbor, Michigan, USA). Absorbance was measure by an ELISA reader (Awareness Technology, Inc., Palm City, USA). Results are given in U/mL.

### 2.7. Hematoxylin and eosin (H&E) 

Tissue samples were cut transverse and fixed in 10% neutral formaldehyde for 48 h. After routine tissue processing, paraffin blocks were obtained. Then, 5 μm serial sections were stained with Haematoxylin and eosin. Microscopic evaluation was performed for healthy under an Olympus BX 51 microscope. Tissue injury in the intestinal mucosa was evaluated using light microscopy according to the criteria described by Guneli et al. graded from 0 to 5 (Table). Histological sections were scored in a blinded, semiquantitative manner using an established scoring scale. For each rat in the groups, at least 10 high power (×1000) fields were examined. The percentage of intestine that displayed massive subepithelial detachments, denudes villi and ulceration was scored as follows 0 = normal mucosa, 1 = slight, 2 = moderate, 3 = massive subepithelial detachments, 4 = denudes villi, 5 = ulceration [11].

**Table:** Scoring system of tissue I/R injury in the intestinal mucosa.

Grade 0	Healthy mucosal villi
Grade 1	Mild neutrophil density in lamina propria, formation of subepithelial detachments at the tip of the villi with capillary congestion
Grade 2	Subepithelial detachments exerted a moderate amount of upward push on the mucosa epithelium, moderate leukocyte density
Grade 3	Large subepithelial detachments exerted a massive amount of upward push on the mucosa epithelium along the villi and few denuded villus tips were observed
Grade 4	The villi were denuded to the level of lamina propria and dilated capillaries
Grade 5	Presence of ulceration, disintegration of lamina propria, and hemorrhage.

### 2.8. Evaluation of intestinal tissues by TUNEL method

Intestinal tissues were isolated for observing TUNEL reaction. Tissues were placed in 10% formaldehyde for 48 h. Paraffin blocks were prepared after the histological process after fixation. Six sections of 4 µm thickness were taken from the paraffin block belonging to each group. “Terminal deoxynucleotidyl transferase-mediated dUTP nick and labeling” (TUNEL) method was used to determine apoptotic cells in sections. TUNEL method was applied as specified in the datasheet of the kit (ApopTag Plus Peroxidase in Situ Apoptosis Kit, S7101, Sigma-Aldrich, St. Louis, MO, USA). Sections were evaluated by a single histologist without knowledge of the distribution of groups. 10 different areas were examined randomly from each section. The number of apoptotic cells in each area examined was evaluated with a computer-based Olympus BX51 microscope and BAB Bs200pro software.

### 2.9. Statistics

Statistical analysis was performed by using GraphPad6 software. The data were analyzed by the Kruskal–Wallis test or one-way analysis of variance with a post hoc Tukey’s or Dunn’s test. Differences with p-values < 0.05 were considered significant. Data are presented as the mean or median ± standard error of the mean or median (25%, 75%).

## 3. Results

### 3.1. Gene expressions of proinflammatory cytokines

**Figure 1:**
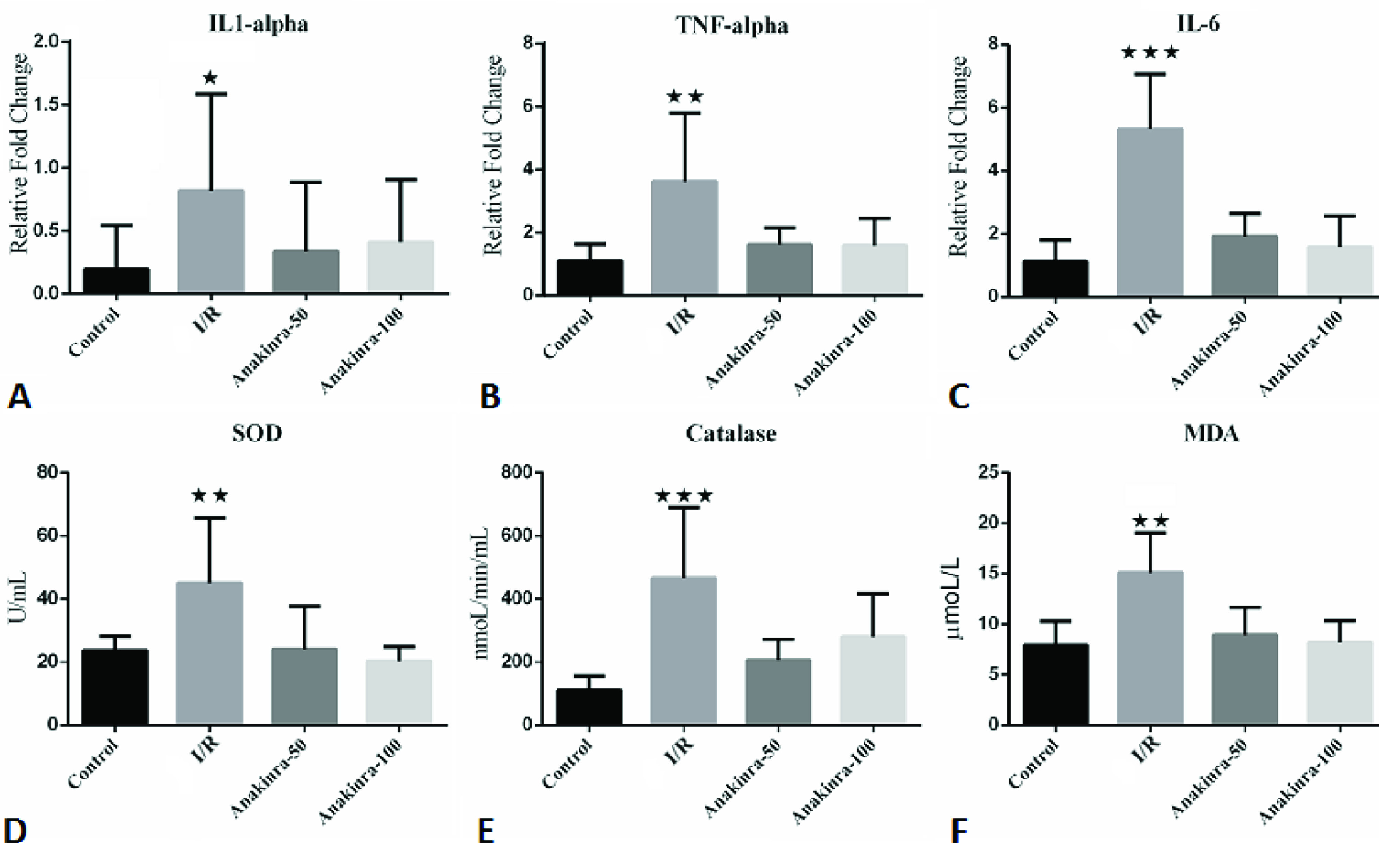
Anakinra attenuates inflammatory response and oxidative stress caused by intestinal I/R injury. Effects of anakinra on (A) IL-1β, (B) TNF-α, (C) IL-6, (D) SOD, (E) CAT, and (F) MDA after intestinal I/R. ***p < 0.001 vs. all other groups; **p < 0.01 vs. all other groups

mRNA levels of proinflammatory cytokines, IL-1α, IL-6, and TNF-α, were significantly higher in the I/R group 2 h after the reperfusion period following ischemia compared with the control group (IL-1α, p < 0.05; IL-6, p < 0.0001; TNF-α, p < 0.01). IL-1α, IL-6 and TNF-α levels were less in groups 3 and 4, in which anakinra was administered than group 2. There was no difference between groups 3 and 4 (Figure 1A, Figure 1B, Figure 1C). 

### 3.2. Measurement of plasma SOD, CAT and MDA levels

Parallel to the cytokine and caspase-3 levels, SOD, CAT, and MDA levels were significantly higher in the I/R group after the reperfusion period compared with the control group (SOD, p < 0.01; CAT, p < 0.001 and MDA, p < 0.01); whereas, all three parameters were significantly decreased in the treatment groups (groups 3 and 4), in which anakinra was administered, than group 2. There was no statistical difference between the treatment groups (Figure 1D, Figure 1E, Figure 1F).  

### 3.3. TUNEL staining

**Figure 2:**
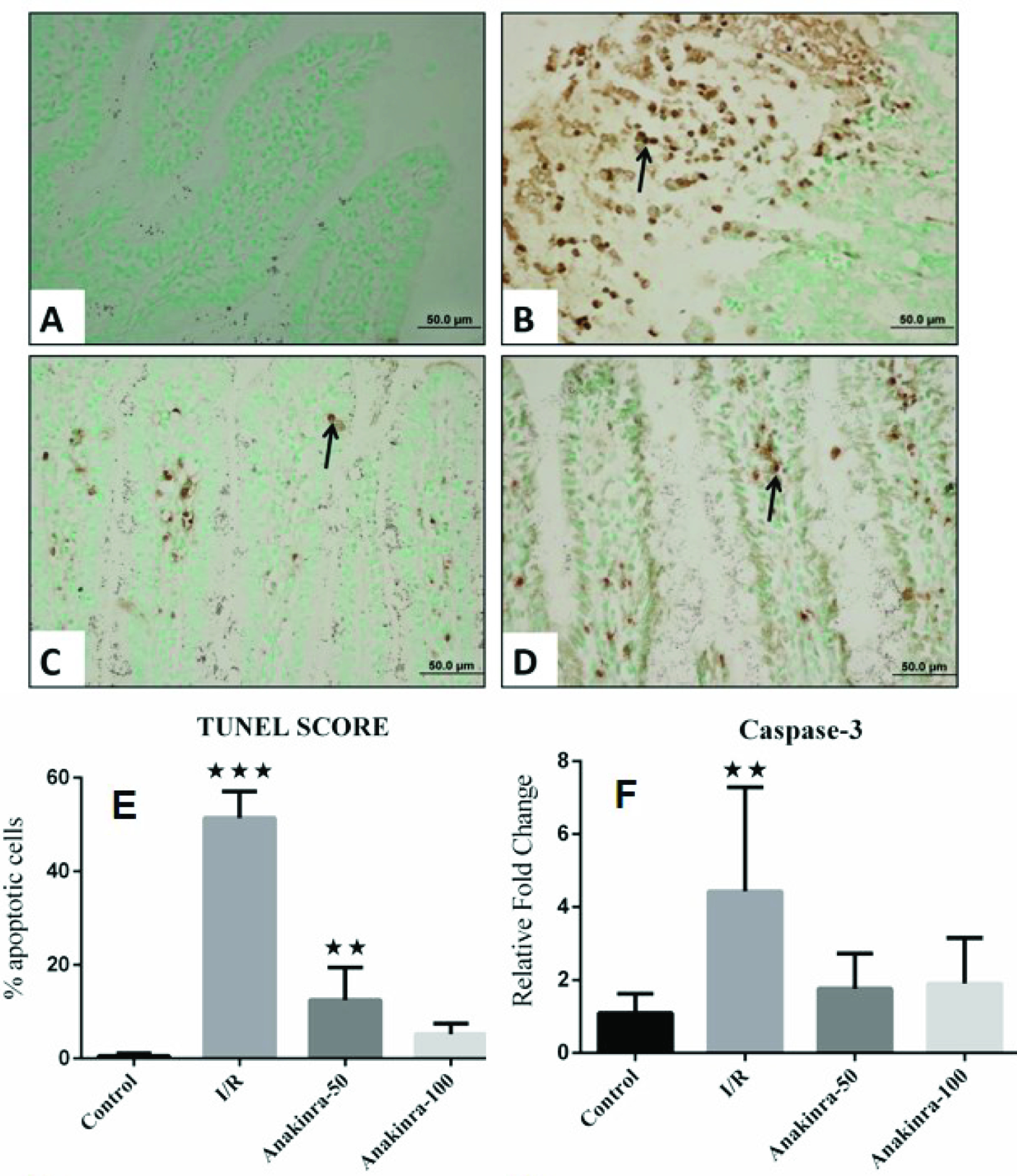
Apoptotic cells in (A) group 1 (control group); (B) group 2 (I/R group); (C) group 3 (treatment group - 50 mg/kg of Anakinra); (D) group 4 (treatment group - 100 mg/kg of Anakinra); (E) Tunel score (% apoptotic cells) (***p < 0.001 vs. all other groups, **p < 0.01 vs. control group); (F) The tissue mRNA levels of caspase-3 (**p < 0.01 vs. all other groups)

To examine the apoptotic cells caused by the occurrence of DNA fractures, we applied the TUNEL method to 6 different sections of each group and obtained images in 10 random areas from each section. When apoptotic cell count was performed on the images in 40x magnifications and the percentage change was examined, the number of apoptotic cells increased by 50.9% in I/R group compared to the control group (Figure 2A, Figure 2B). After ischemia/reperfusion, the number of apoptotic cells in the group treated with 50 mg/kg Anakinra was 15.8%, and in the group that received 100 mg/kg Anakinra, the apoptotic cells were found to be 5% (Figure 2C, Figure 2D). While negative reactions were observed mostly in the intestinal samples belonging to the control group (Figure 2A), in the I/R group that we created I/R model, we observed apoptotic cells that showed a high rate of positive reaction in the cells pouring into lumen (Figure 2B). When 50 mg/kg Anakinra is applied to rats with I/R model, apoptotic cell number and reaction severity (Figure 2C) show a significant decrease in preserved villi structures compared to I/R group. When 100 mg/kg Anakinra is applied to rats with I/R model, very few apoptotic cells are detected in prominent villi structures (Figure 2D). As a result, the TUNEL score was greatest in the I/R group which was reduced in the treatment groups (Figure 2E).

### 3.4. Gene expression of caspase-3

mRNA level of caspase-3 was significantly higher in the I/R group 2 h after the reperfusion period following ischemia compared with the control group (p < 0.01). Caspase-3 mRNA levels were less in groups 3 and 4, in which anakinra was administered than group 2. There was no difference between groups 3 and 4 (Figure 2F). 

### 3.5. Hematoxylin and eosin staining

**Figure 3:**
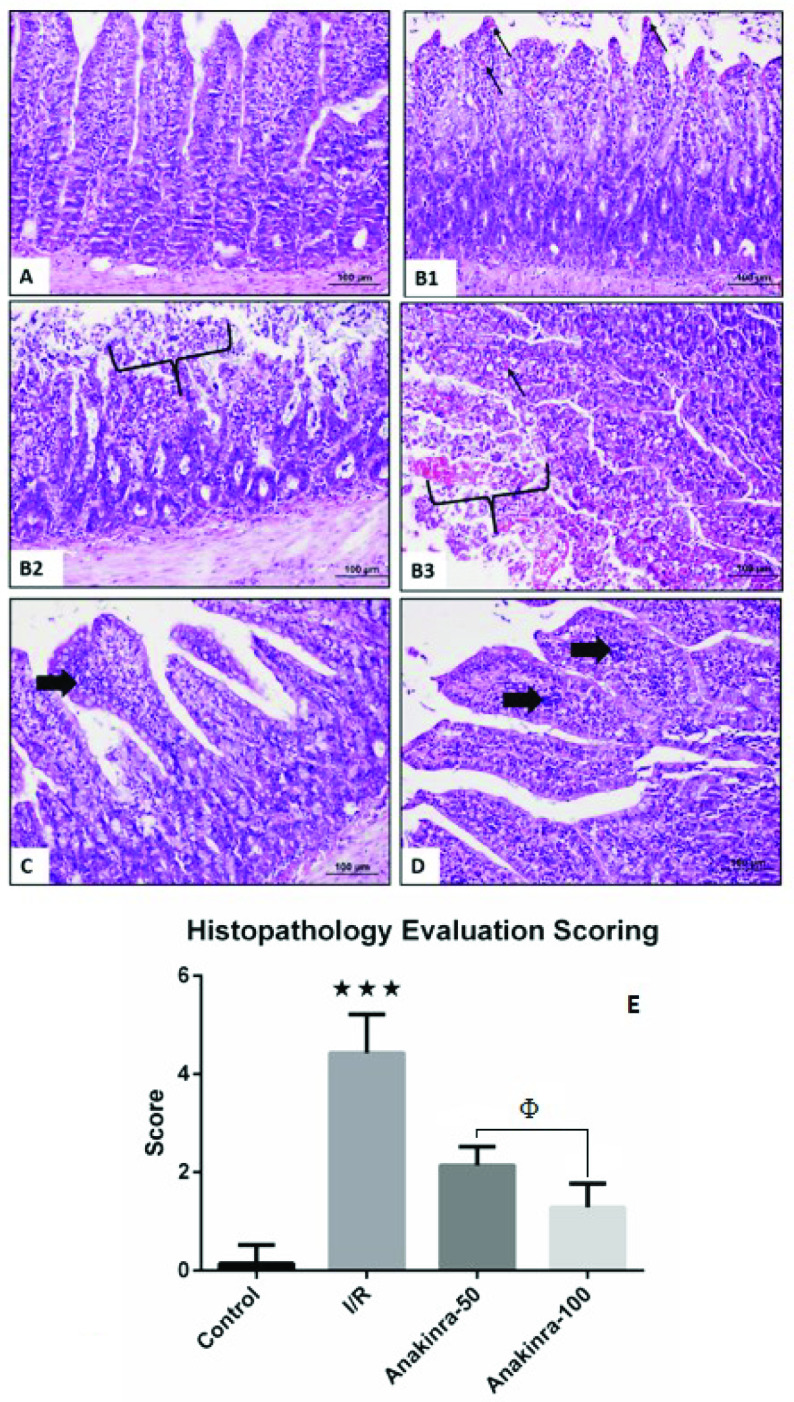
Histological examination of changes in villi structures in intestinal tissues (HE, x200). Group 1 in which healthy villus structures were observed (Control group, A). Group 2 (I/R group, B, C, D) in which no goblet cells are observed and capillary congestion is detected at the apex of the villi (thin arrow), the lamina propria is spilled (parenthesis). Group 3 with lymphocyte foci (thick arrow) in the lamina propria (treatment group - 50 mg/kg of Anakinra, E). Group 4 with decreased lymphocyte foci (thick arrow) in the lamina propria (treatment group - 100 mg/kg of Anakinra, F). (G) Histopathology evaluation scoring (***p < 0.001 vs. all other groups; Φp < 0.01, treatment group - 50 mg/kg of Anakinra vs. treatment group - 100 mg/kg of Anakinra).

Healthy villi structures consisting of eosinophilic cytoplasm and intestinal epithelial cells with euchromatin nuclei and goblet cells were observed in the control group (Grade 0) (Figure 3A). Regions of different grades were observed in the intestinal samples belonging to the ischemia group. The connection units forming the epithelium have been disrupted and therefore the villi structure has been completely lost, the epithelial tissue and the lamina propria have been mostly spilled (Grade 5) (Figure 3B, Figure 3C, Figure 3D). The villi were denuded to the level of lamina propria and dilated capillary is remarkable in the lamina propria (Grade 4) (Figure 3C, Figure 3D). In I/R rats with 50 mg/kg anakinra, the villi structure was partially preserved, but increased leukocyte density was found in the lamina propria (Grade 2) (Figure 3E). Although the villus structures of IR rats administered 100 mg/kg anakinra were healthier than those administered 50 mg/kg anakinra, mild neutrophil density was observed in lamina propria (Grade 1) (Figure 3F).

## 4. Discussion

Intestinal I/R injury is a clinical entity, which not only damages the intestinal tissue but also results in a systemic inflammatory response which may increase mortality [4,12]. Intestinal I/R damage has continuously been investigated since there is still no definitive therapy. In the present study, our main finding was that anakinra, an IL-1 receptor antagonist could attenuate intestinal I/R damage by reducing the inflammatory response.

It has been reported that the levels of proinflammatory cytokines such as IL-1, IL-6, and TNF-α, which are crucial for PMN migration to the ischemic site, can be reduced by anakinra as in our study [13,14]. The relative fold changes in IL-6 and TNF-α levels were somewhat greater than IL-1 levels following I/R. We assume that the increase in IL-1 levels triggered the inflammatory cascade which had resulted in greater fold changes in the levels of IL-6 and TNF-α. The antagonist of IL-1 receptor not only antagonizes the signal transduction of IL-1 but also blocks the synthesis and action of downstream inflammatory mediators of IL-1, such as IL-6 and TNF-α [13]. Probably, the additive effects of the blockage of the inflammatory cascade and the direct suppression of the synthesis of IL-6 and TNF-α are the reasons for the greater reduction in the levels of IL-6 and TNF-α compared to the reduction in the level of IL-1α in anakinra treatment groups. 

Free radicals that are generated by activated PMNLs during reperfusion contribute to the I/R injury. SOD eliminates superoxide anions by converting superoxide anions into hydrogen peroxide which is then removed by CAT. Thereby, SOD and CAT indicate the level of oxidative stress level [5]. Hasturk et al have shown that SOD levels were decreased as well as CAT and MDA levels as a sign of reduction of oxidative stress in the anakinra treatment groups in animal models of brain injury and spinal cord I/R injury in parallel with the reduction in tissue IL-1β levels [15,16]. In the study of Jin et al, intestinal MDA levels were decreased following IL-1 receptor antagonist (IL-1Ra) treatment, which they prepared using the recombinant plasmid pBV220/IL-1Ra, in a model of intestinal ischemia as in our study [17]. However, in contrast to our findings, intestinal SOD levels were elevated in IL-1Ra treatment groups and they proposed that inhibition of IL-1 receptors increased the activity of SOD in the damaged tissue. However, our results were more consistent with the findings of the studies of Hasturk et al. We observed that anakinra reduced the production of SOD and CAT by inhibiting the recruitment of leukocytes to the ischemic site as we have shown histopathologically. 

It has been demonstrated that oxidative stress in I/R injury can induce apoptosis as shown in cardiac and ovarian models [8,18]. Similarly, we showed increased apoptosis as a result of oxidative stress in intestinal I/R. In these I/R models, anakinra performed protective effects. In the study of Nayki et al on an ovarian model of I/R, anakinra decreased caspase-3 expression and ameliorated oxidative induced apoptosis more effectively at a dose of 100 mg/kg than 50 mg/kg [18]. In our study, caspase-3 expression was greater in the I/R group than in the control group and treatment groups whereas there was no difference between the control group and treatment groups. On the other hand, TUNEL-positive cell numbers were less in the treatment groups compared to the I/R group and the difference between the control group and group 3 was diminished in group 4. These data suggest that anakinra may reduce intestinal cell apoptosis and is more effective at a dose of 100 mg/kg. Our histopathological findings also supported this proposal since the villous structure of the small intestine was better preserved at a dose of 100 mg/kg than 50 mg/kg. 

In conclusion, anakinra decreased the intestinal damage caused by I/R and a dose of 100 mg/kg was found to be more effective. These results indicate that anakinra might be useful in clinical practice to treat the intestinal I/R injury but need to be verified by larger animal studies and appropriate clinical studies.
